# 20 years of daily hospital for non-psychotic disorders, organisational structure and treatment modalities

**DOI:** 10.1192/j.eurpsy.2022.1940

**Published:** 2022-09-01

**Authors:** S. Caratan, S. Kocijan Lovko, A. Ivrlač, L. Goršić, T. Matoš

**Affiliations:** 1 University Psychiatric Clinic “Sveti Ivan”, Day Hospital For Non-psychotic Disorders, Zagreb, Croatia; 2 Faculty of Dental Medicine and Health Osijek, Josip Juraj Strossmayer University of Osijek, Department Of Psychiatry, Osijek, Croatia; 3 University Psychiatric Clinic “Sveti Ivan”, Day Hospital For Non-psychotic Disorders, zagreb, Croatia

**Keywords:** Daily hospital, anxiety, depression, treatment

## Abstract

**Introduction:**

Anxiety and depressive disorders are among the most prevalent psychiatric disorders, yet there aren’t many studies adressing treatment modalities in daily hospitals.

**Objectives:**

The aim of our study is to present organisational structure and treatment modalities in Daily hospital for non-psychotic disorders in University Psychiatric Clinic “Sveti Ivan“, Zagreb, Croatia.

**Methods:**

All patients in Daily Hospital attend 6 months programme. The treatment programme consists of psychodynamic group psychotherapy, cognitive-behavioral therapy, occupational therapy, antistress and mindfulness workshops, psychoeducational workshops, cinema therapy and sociotherapy. Along with clinical follow-up, all the patients are given sociodemographic questionnaire designed for our programme, World Health Organisation Quality of Life Questionnaire (WHOQoL BREF) and The Depression, Anxiety and Stress Scale 21 (DASS-21).

**Results:**

At discharge moderate to significant improvement was observed in different aspects of functioning among most of the participants, verified by clinical follow-up as well as by results obtained through questionnaires.

**Conclusions:**

Our data show that patients with anxiety and depressive disorders can be efffectively treated by such comprehensive treatment approach.

**Disclosure:**

No significant relationships.

